# Treatment and prognosis of type B2 thymoma

**DOI:** 10.1186/1477-7819-12-291

**Published:** 2014-09-20

**Authors:** Zhengbo Song, Xiangyu Jin, Yiping Zhang

**Affiliations:** Department of Chemotherapy, Zhejiang Cancer Hospital, 38 Guangji Road, Hangzhou, 310022 China; Key Laboratory Diagnosis and Treatment Technology on Thoracic Oncology, Hangzhou, Zhejiang Province 310022 China

**Keywords:** thymoma, type B2, treatment, prognosis

## Abstract

**Background:**

Because of the rarity of thymoma, no randomized trials have prospectively detected the prognosis and optimal treatment for type B2 thymoma. The aim of this study is to investigate the treatment and prognosis for patients with type B2 thymoma.

**Methods:**

We retrospectively evaluated the outcome of 42 patients with type B2 thymoma who were treated between 1995 and 2010 in Zhejiang Cancer Hospital. Survival curves were plotted using the Kaplan–Meier method. The Cox proportional hazard model was used for multivariate analysis.

**Results:**

The current study included 42 patients. The 5-year disease-free survival and overall survival rates were 62.8% and 84.9%, respectively. There were significant differences in disease-free survival and overall survival among different Masaoka stages (both *P* < 0.001). Univariate and multivariate analysis revealed that Masaoka stage was associated with disease-free survival and overall survival (*P* = 0.00 and 0.001, respectively).

**Conclusions:**

Our results indicate that Masaoka stage affects disease-free survival and overall survival of patients with type B2 thymoma.

## Background

Thymoma has a prevalence of 0.1 to 0.4/100,000 and accounts for less than 1% of all malignant tumors [1]. The B2 type of thymoma is defined as a tumor in which the neoplastic epithelial component appears as scattered plump cells with vesicular nuclei and distinct nucleoli among a heavy population of lymphocytes by the World Health Organization (WHO) classification [2].

The proportion of B2 types reported is about 20% for all thymomas [3, 4]. The B2 type of thymoma has been shown to have a better prognosis than thymic carcinoma and worse than types A, AB and B1 [5, 6]. Because of the rarity of thymoma, no randomized trials have prospectively detected the prognosis and optimal treatment of type B2 thymoma. No consensus guidelines for the treatment of type B2 thymoma have currently been defined. In the present study, we retrospectively evaluated the treatment and prognosis of patients with type B2 thymoma, and the long-term efficacy of multimodality therapy in this setting.

## Methods

### Patients

A total of 252 patients underwent a surgical resection for thymoma between January 1995 and January 2010 in Zhejiang Cancer Hospital. In this period, 42 patients were diagnosed as type B2. The stage of thymoma was classified based on the Masaoka staging system. All patients had been pathologically confirmed as having type B2 thymoma (WHO histologic classification) by surgery or needle biopsy. Recurrence or metastases were confirmed using chest computed tomography, as well as ultrasound and/or computed tomography of the abdomen. The study was approved by the ethics committee of Zhejiang Cancer Hospital.

### Follow-up

For patients who underwent a surgical intervention, all were examined in the outpatient clinic at 3-month intervals for the first 2 years and, thereafter, at 6-month intervals. For patients at an advanced stage, the follow-ups were at 6 to 8 weeks apart. The last censoring date for survival was March 2013. The median follow-up of patients was 70 months, ranging from 25 to 180 months.

### Statistical analysis

The statistical analysis was performed using SPSS version 17 (SPSS Inc, Chicago, IL, USA), assuming that *P* < 0.05 is statistically significant. The survival curves were generated using the Kaplan–Meier method. Disease-free survival (DFS) encompassed the time from surgery to documented progression or death from any cause. The definition of overall survival (OS) was determined from the date of surgery and the last known follow-up or date of death.

## Results

### Patient characteristics

The patients’ characteristics are detailed in Table 1. The median age was 54 years (range 15 to 71 years). Of the 42 patients, 35 underwent surgery and 7 received a biopsy. At the time of diagnosis, 31 were asymptomatic, while 11 presented with clinical symptoms or signs, including 8 with myasthenia gravis and 3 with chest pain. Stage I was diagnosed for 14 patients, stage II for 11 patients, stage III for 10 patients and stage IV for 7 patients.

**Table 1:** **Characteristics of the 42 patients**

Variable	Number	Percentage
Sex		
Male	16	38.1
Female	26	61.9
Age		
Median (years)	54	
≥50	25	59.5
<50	17	40.5
Tumor size		
>7 cm	11	26.2
≤7 cm	31	73.8
Myasthenia gravis		
Yes	8	19.0
No	34	81.0
Masaoka stage		
I	14	33.3
II	11	26.2
III	10	23.8
IV	7	16.7
Surgery		
R0	30	71.4
R1 + R2	5	11.9
No	7	16.7
Radiotherapy		
Yes	22	52.4
No	20	47.6
Chemotherapy		
Yes	6	14.3
No	36	85.7

### Treatment and recurrence or metastasis

Of the 35 patients who underwent surgery, 30 had an R0 resection and 5 had an R1 or R2 resection. Also 22 patients were treated with radiation therapy (doses ranged from 40 to 60 Gy). Six patients received chemotherapy as a regimen with CAP (cyclophosphamide + doxorubicin + cisplatin, *n* = 2), carboplatin and paclitaxel (*n* = 2) or VIP (ifosfamide + cisplatin + etoposide, *n* = 2).

Recurrence developed in 17 patients. Ten of seventeen patients had local recurrence, and pleural relapse was the most common site (six of ten patients). Seven patients had distant metastases after surgery. The sites of the distant metastases were the lung (three cases), liver (two cases) and bone (two cases).

### Survival and prognostic factors

The 5-year DFS rates were 62.8% (85.7%, 70.1%, 47.3% and 0.00% in stages I, II, III and IV, respectively). The 5-year OS rates were 84.9% (91.7%, 88.9%, 72.0% and 34.3% in stages I, II, III and IV, respectively).

**Figure 1:**
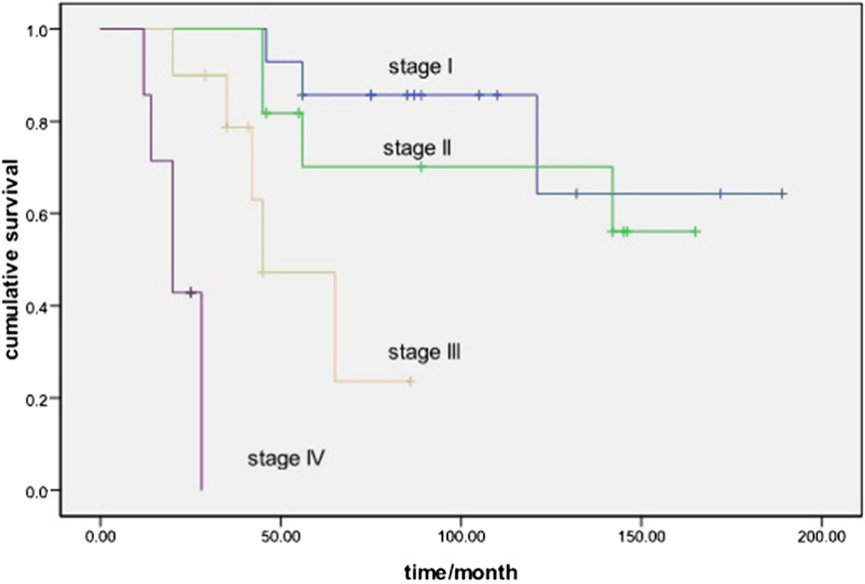
**Comparison of disease-free survival for different Masaoka stages (** ***P*** **< 0.001).**

**Figure 2:**
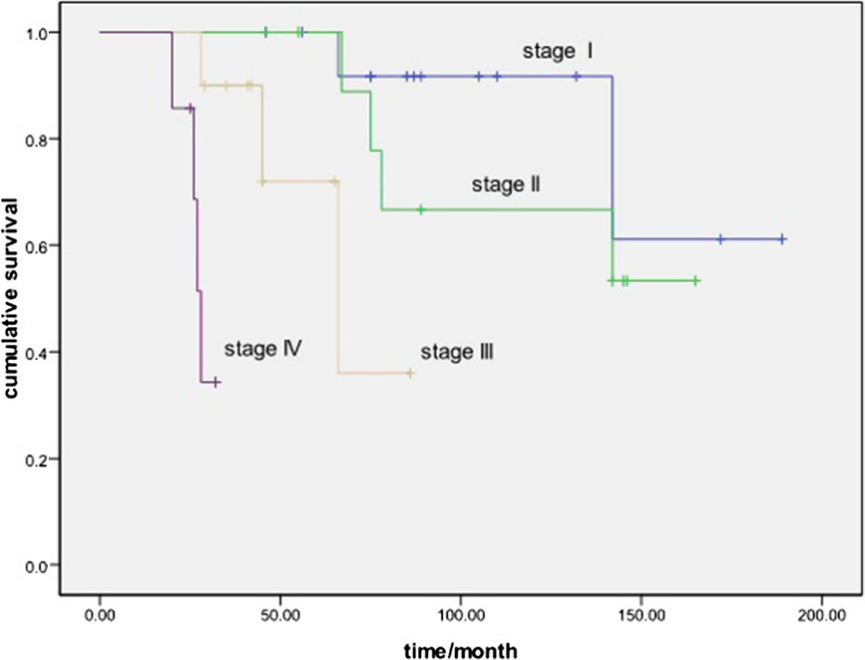
**Comparison of overall survival for different Masaoka stages (** ***P*** **< 0.001).**

The effects of age, gender, adjuvant treatment, myasthenia gravis, resection and Masaoka stage on DFS and OS were studied in both uni- and multivariate analyses (Tables 2 and 3). In the univariate analysis, resection and Masaoka stage were predictive of DFS and OS (Figures 1 and 2). Masaoka stage was shown to have a significant influence on DFS and OS in the multivariate analysis.

**Table 2:** **Univariate analysis of disease-free and overall survival for 42 patients with type B2 thymoma**

Variable	Five-year disease-free survival rate (%)	***P***	5-year overall survival rate (%)	***P***
Gender		0.117		0.061
Male	44.9		87.5	
Female	75.1		82.8	
Age		0.479		0.971
<50	62.1		87.7	
≥50	62.9		81.4	
Stage		0.000		0.000
I + II	79.2		95.2	
III + IV	33.4		55.2	
Resection		0.009		0.000
Yes	67.8		93.7	
No	42.9		51.4	
Adjuvant treatment		0.204		0.023
Yes	66.5		95.7	
No	58.9		71.5	
Tumor size		0.772		0.372
>7 cm	56.2		90.7	
≤7 cm	80.8		82.4	
Myasthenia gravis		0.895		0.702
Yes	51.4		83.3	
No	64.7		84.9

**Table 3:** **Multivariate survival analysis for disease-free and overall survival of the 42 patients**

		Disease-free survival			Overall survival	
Variable	Hazard ratio	95% confidence interval	***P***	Hazard ratio	95% confidence interval	***P***
Gender (male vs female )	0.448	0.144–1.395	0.166	0.275	0.069–1.100	0.068
Age (≥50 vs <50)	1.058	0.249–4.507	0.939	0.777	0.134–4.491	0.778
Resection (yes vs no)	0.782	0.113–5.429	0.804	1.937	0.219–17.092	0.552
Adjuvant treatment (yes vs no)	1.385	0.421–4.555	0.591	2.853	0.649–12.545	0.165
Tumor size (>7 cm vs ≤7 cm)	3.079	0.657–14.417	0.153	2.514	0.371–17.041	0.345
Myasthenia gravis (yes vs no)	1.204	0.182–7.945	0.847	0.598	0.046–7.813	0.695
Stage (III + IV vs I + II )	6.163	2.421–15.691	0.000	5.462	1.923–15.514	0.001

## Discussion

To the best of our knowledge, this is the first report that has focused on the prognosis and treatment of type B2 thymoma. Our results suggest that Masaoka stage could affect the survival of patients with type B2 thymoma.

Type B2 thymoma has a moderate aggressive nature compared with other subtypes. About half of patients were staged as III or IV at the first diagnosis [7, 8]. In contrast, most patients with type B3 and thymic carcinoma were at an advanced stage [9, 10]. In the current study, 40.5% patients were at stages III or IV of type B2 at diagnosis. The 5-year survival rate was around 50% for type B3 thymoma, while it was 90% for types B1, AB and A. The 5-year survival rate for type B2 thymoma was about 60% to 70% in previous reports [4, 5]. The 5-year survival rate was 62.8% in our study, which is similar to previous reports.

The effect of different treatments is discussed controversially in previous studies. It is generally accepted that surgery is the mainstay treatment, and R0 resection is one of the most independent prognostic factors for thymoma. However, some reports have shown that there was no survival difference for patients who undergo complete resection compared with patient undergoing incomplete resection [4, 11]. In the current study, there were DFS and OS differences between complete and incomplete resection patients. However, the differences for DFS and OS were not significant by multivariate analysis, possibly because of the small number of patients in our study. Adjuvant radiotherapy is not usually recommended for stage I thymoma in the National Comprehensive Cancer Network guideline and recommended category is low in stage II and III thymoma. In our study, adjuvant radiotherapy and chemotherapy had no effect on DFS and OS in multivariate analyses. Considering selection bias and the small sample size, no conclusions can be made for the efficacy of adjuvant treatment.

The Masaoka staging system has been shown to be an independent prognostic factor for all subtypes of thymoma in previous research [12, 13]. Our present study demonstrated that Masaoka stage was an independent prognostic factor for type B2 thymoma in both uni- and multivariate analyses (*P* = 0.00 and 0.001, respectively). There were significant survival differences among different stages in terms of DFS and OS (Figures 1 and 2).

Our study is limited by its retrospective design and its small number of patients. In addition, there were only 17 patients with recurrence or metastasis. Therefore, the outcome must be explained carefully. However, with few cases in previous clinical studies, our retrospective study may also be considered to be meaningful.

## Conclusions

In conclusion, Masaoka stage remains the mainstay for type B2 thymoma. Given the nature of retrospective analysis, our results should be confirmed by further prospective studies.

## Consent

Written informed consent was obtained from the patient for the publication of this report and any accompanying images.

## Abbreviations

DFS: disease-free survival
OS: overall survival
WHO: World Health Organization.
